# Construction of a high density linkage map in Oil Palm using SPET markers

**DOI:** 10.1038/s41598-020-67118-y

**Published:** 2020-06-19

**Authors:** Javier Herrero, Baitha Santika, Ana Herrán, Pratiwi Erika, Upit Sarimana, Fahmi Wendra, Zulhermana Sembiring, Dwi Asmono, Enrique Ritter

**Affiliations:** 1 NEIKER-Basque Institute for Agricultural Research and Development - Basque Research and Technology Alliance (BRTA). Campus Agroalimentario de Arkaute s/n, 01192 Arkaute, Spain; 2 Department of Research & Development, PT Sampoerna Agro Tbk., Jl. Basuki Rahmat No. 788, Palembang, 30127 Indonesia

**Keywords:** Next-generation sequencing, Genetic markers, Plant breeding, Plant genetics, Plant breeding, Plant domestication

## Abstract

A high-density genetic linkage map from a controlled cross of two oil palm (*Elaeis guineensis*) genotypes was constructed based on Single Primer Enrichment Technology (SPET) markers. A 5K panel of hybridization probes were used for this purpose which was derived from previously developed SNP primers in oil palm. Initially, 13,384 SNPs were detected which were reduced to 13,073 SNPs after filtering for only bi-allelic SNP. Around 75% of the markers were found to be monomorphic in the progeny, reducing the markers left for linkage mapping to 3,501. Using Lep-MAP3 software, a linkage map was constructed which contained initially 2,388 markers and had a total length of 1,370 cM. In many cases several adjacent SNP were located on the same locus, due to missing recombination events between them, leading to a total of 1,054 loci on the 16 LG. Nevertheless, the marker density of 1.74 markers per cM (0.57 cM/marker) should allow the detection of QTLs in the future. This study shows that cost efficient SPET markers are suitable for linkage map construction in oil palm and probably, also in other species.

## Introduction

One of the most productive oil crops in the world is the oil palm. According the USDA (United States Department of Agriculture), the total world vegetable oil production of 2019/2020 (until December 2019) was 207.06 million MT, with palm oil in the first place (75.69 million MT or 36.6%), followed by soybean oil (56.73 Million MT) and rapeseed oil (27.04 Million MT). Oil palm (*Elaeis guineensis* Jacq.) is a perennial monocotyledonous tropical crop species that belongs to the family of *Arecaceae*, which originate from the tropical rain forest of Central and West Africa.

Conventional breeding based on phenotypic observations in the progenies is generally applied in oil palm breeding. However, it needs more space and time for selecting promising crosses, particularly when increasing parental biodiversity. Since the land issues are spreading^1^, breeders are starting slowly to implement molecular breeding techniques for improving the oil palm, both production and quality, without enlarging more the land use.

Several possibilities for genetic material selection and improvement in *E. guineensis* using molecular breeding have been proposed by several authors^2–4^. One way to overcome these issues consists of optimizing the molecular breeding through marker assisted selection^5,6^. Selection of promising parental palms can be done already at seedling stage, without waiting for the palm to give information about production. Marker assisted selection (MAS) can also reduce the land use based on progeny performance predictions and earlier selection during the nursery step, reducing in this way the number of progenies for evaluations. MAS usually requires prior knowledge on the distribution of quantitative trait loci (QTL) for a targeted trait in the genome and the underlying candidate genes^7^.

Large number of genomic resources has been generated in the last decades for this purpose in oil palm, including the whole genome sequence. These resources can be accessed at the National Center of Biotechnology (NCBI, USA) in the Taxonomy database (http://www.ncbi.nlm.nih.gov/Taxonomy/) or at PalmXplore from Malaysian Palm Oil Board (MPOB)^8^ (http://palmxplore.mpob.gov.my/palmXplore). Also different molecular and statistical techniques have been applied in oil palm in molecular breeding, such as Genome wide Association studies (GWAS) based on GBS “genotyping by sequencing” for candidate gene detection^9^ or Genomic Preselection based on GBS for breeding^10^. These techniques were applied on whole germplasm collections.

In the past, a more classical way of molecular breeding represented the construction of genetic linkage maps in controlled crosses and posterior QTL analyses. Markers close to the QTL locations could be applied for MAS, and if the map was anchored to a physical map, QTL locations could be searched for potential co-located candidate genes with a relevant biological meaning influencing a particular trait.

Various examples are available in oil palm. For example, Billotte *et al*.^11^ performed QTL detection for different productive and oil quality traits by multi-parent linkage mapping in oil palm. Montoya *et al*.^12^ identified 19 QTL related to fatty acid composition in an interspecific pseudo-backcross between *E.oleifera* and *E. guineensis* and Seng *et al*.^13^ mapped QTLs for oil yield components in an elite oil palm cross.

Despite more sophisticated analysis methods which are nowadays available, classical linkage mapping and QTL analyses in a controlled cross represents still a valid and useful tool in the case of rare genotypes with exceptional trait expression, as for example a rare resistance to a particular disease, for which no collection of genotypes with varying resistance levels are available.

Different marker types have been used widely in oil palm for constructing linkage map which include RFLPs, AFLPs, SSRs, and SNPs. Restriction associated DNA tagging (RAD) or double digestion RAD (ddRAD) have identified large amount of SNPs and produced remarkable maps^14,15^. These are low cost approaches for linkage mapping, relying on target sequences between restriction sites all over the genome, including coding and non-coding regions. Recently, specific sequence capture (probe based) methods have become also a cost effective alternative. Since only a small amount of gDNA from each sample has to be provided beside target genome and SNP information, affordable sequencing is also possible for modest laboratories. A 5K probe panel should ensure a good coverage of target loci and the detection of polymorphisms in the mapping population^16^. The single primer enrichment technology (SPET), recently developed by NUGEN, is a targeted sequencing technology which has been used up to now for biomedical applications^17,18^ and in plants for genotyping in monocot (*Z. mays*) lines or in a natural black poplar (*P. nigra*) population^19^, as well as for characterizing large germplasm sets from tomato and egg plant^20^. According to Barchi *et al*.^20^, SPET represents a valid alternative to GBS and micro arrays, and it allows users to customize the panel of target markers and provides reliable fingerprinting of accessions maintained in gene banks. Also for linkage mapping, specific capture ensures the detection of existing population polymorphisms at all targeted positions. The scalable probe design can maximize the number of target locations and the sequencing of all SNPs in the genomic regions for which probes have been designed. Thus, this technique appears to be especially well suited for the construction of dense genetic maps^16^.

Our main objective was to provide a cost-effective alternative for obtaining a sufficiently saturated linkage map using own genetic resources, a small sample number and considering a limited laboratory infrastructure. This study tests the suitability of the novel SPET technology for constructing a high-density linkage map which has been never used before in oil palm and can be further exploited for QTL analyses and breeding in the future.

## Results

The service provider delivered a VCF file with a total of 13,384 SNP markers located on all 16 chromosomes of oil palm. The SNP distribution over chromosomes and their correspondence to individual loci was analysed. Table 1 summarizes the results. The SNP numbers per chromosome varied from 443 to 1634 with an average of 836.5 SNP per chromosome. They corresponded to a total of 4308 (out of 5000) loci, where each locus represented a designed SPET probe. The corresponding loci varied from 150 to 537 with an average of 269.3 loci per chromosome. In accordance, the average numbers of detected SNP per probe ranged from 2.89 to 3.44, and on average 3.12 SNP per probe were observed. However, in many cases 5 or more SNP per locus were detected. For the probe design of each SNP the OPGP reference genome sequence of dura DELI genotype PO4906D was used. The frequently observed additional SNP resulted from quite different sequences in the more exotic parental genotypes from Cameroon and Nigeria at the SNP locations.

**Table 1:** Distribution of SNP over chromosomes and number of probe loci.

CHR	No of SNP	No of Loci	SNP/Locus
CHR1	1193	367	3.25
CHR2	836	273	3.06
CHR3	589	171	3.44
CHR4	1634	537	3.04
CHR5	482	151	3.19
CHR6	893	266	3.36
CHR7	565	176	3.21
CHR8	1299	443	2.93
CHR9	690	231	2.99
CHR10	779	260	3.00
CHR11	786	251	3.13
CHR12	922	319	2.89
CHR13	629	201	3.13
CHR14	631	202	3.12
CHR15	1013	310	3.27
CHR16	443	150	2.95
**Sum**	**13384**	**4308**	**—**
**Mean**	**836.5**	**269.3**	**2.29**

CHR: chromosomes, No of Loci: number of loci targeted by individual hybridization probes, SNP/Locus: detected SNP per targeted probe locus.

SNPs were filtered for a maximum of 20% missing values. A total of 13,256 SNPs remained. SNPs were also filtered for bi-allelic states. A total of 183 SNPs showed more than 2 allelic states, leading to 13,073 remaining SNPs.

After imputing missing values with Beagle 5 Software, we analysed the expected segregations of SNP markers. Table 2 shows the expected segregation ratios depending on the parental configuration (PC). In theory, nine unphased PC are possible. In four cases, no segregation was expected (both parents were homozygous for one SNP level), while in four cases 1:1 segregations occurred (only one parent was homozygous for one SNP allele, the other parent was heterozygous) and in one case a 1:2.1 segregation was expected (both parents were heterozygous). Table 2 shows also the observed numbers of SNP cases for each parental configuration.

**Table 2:** Expected Segregations depending on the parental SNP configurations and observed SNP numbers for each parental configuration.

*Parental Configuration*		*Progeny SNP Marker Classes*			SEG	No SNP
*P1*	*P2*	*F1a*	*F1b*	F1c		
*0/0*	*0/0*	*0/0*	***—***	**—**	noS	8652
***0/0***	***0/1***	***0/0***	***0/1***	**—**	**1:1**	**977**
*0/0*	*1/1*	***—***	*0/1*	**—**	noS	37
***0/1***	***0/0***	***0/0***	***0/1***	**—**	**1:1**	**658**
***0/1***	***0/1***	***0/0***	***0/1***	**1/1**	**1:2:1**	**1063**
***0/1***	***1/1***	***—***	***0/1***	**1/1**	**1:1**	**234**
*1/1*	*0/0*	***—***	*0/1*	**—**	noS	31
***1/1***	***0/1***	***—***	***0/1***	**1/1**	**1:1**	**569**
*1/1*	*1/1*	***—***	***—***	1/1	noS	852
			***Total number of SNP***:			***13073***
			***No of segregating SNP***:			***3501***
			***No of non-segregating SNP***:			***9572***

Reference SNP Allele = 0, Alternative SNP Allele = 1.

Parental or Progeny Genotype Configurations (unphased):

0/0 GT homozygous for Reference Allele.

0/1 GT heterozygous for Reference and Alternative Allele.

1/1 GT homozygous for Alternative Allele.

P1 P2: Parent 1 and 2, F1i: expected SNP marker classes in F1 progeny, SEG: expected segregation ratios of SNP marker classes (noS = no segregation), No SNP numbers of observed SNP with the corresponding parental configuration.

With respect to the classification of SNP depending on the parental configuration, the highest numbers of SNP were homozygous for the reference allele in both parents (P1:0/0-P2:0/0). This was the case for 8,652 SNP (66.2%) for which no segregation was expected. In contrary, for the PC with homozygous states for the alternative allele (1/1-1/1) only 852 cases were found. Considering other PC without expected segregations, remarkable low numbers of cases were observed for the PC: 1/1-0/0 and 0/0-1/1 which occurred in only 31 and 37 cases, respectively. Thus, a total of 9,572 SNP (73.2%) revealed a non-segregating PC. In general, there was a good agreement of the observed and the expected segregations for the segregating PC. The other PC frequencies with expected segregations varied between 234 and 1,063 SNP.

Non-segregating SNPs were discarded from the imputed VCF file and a total of 3,501 SNPs (26.8%) remained for further processing by Lep-MAP. Although several SNP showed high distortions in their segregation ratios, they were kept since Lep-MAP would filter them automatically during the process of linkage map construction. Table 3 summarizes the results obtained by Lep-MAP. Initially 20 linkage groups were obtained. The first 16 linkage groups corresponded to the 16 chromosomes of the oil palm genome. They contained a total of 2,388 markers and had a total length of 1,370 cM. Individual linkage groups contained between 71 and 280 SNP markers each and varied between 57.1 and 154.7 cM in length. This corresponded to an average marker density of 1.74 marker per cM (0.57 cM/marker). In addition, four smaller linkage groups were obtained with a total of 27 markers. These markers belonged to other chromosomes, but could not be placed on the corresponding linkage groups.

**Table 3:** Characteristics of the Linkage Map obtained by Lep-MAP Software.

LG	Mno	Length	LogL	missPlaced	NoM	Loci	devM
1	188	**94.0**	−5494.3	10	**178**	**74**	7(3)
2	111	**85.9**	−2735.9	2	**109**	**58**	6(5)
3	134	**70.4**	−2717.8	1	**133**	**52**	7(4)
4	289	**154.7**	−7157.8	9	**280**	**122**	17(12)
5	106	**64.3**	−1739.2	1	**105**	**53**	4(1)
6	90	**78.8**	−2316.0	12	**78**	**49**	6(3)
7	119	**73.4**	−2684.2	8	**111**	**56**	5(3)
8	268	**128.9**	−9723.0	1	**267**	**114**	19(11)
9	168	**87.9**	−4218.2	14	**154**	**75**	6(4)
10	86	**50.9**	−1628.4	5	**81**	**45**	10(4)
11	166	**113.5**	−5322.2	7	**159**	**76**	9(5)
12	133	**85.4**	−3187.7	2	**131**	**59**	4(2)
13	138	**72.6**	−2817.4	4	**134**	**69**	10(3)
14	126	**66.1**	−2742.8	8	**118**	**48**	0(0)
15	195	**86.4**	−5146.8	6	**189**	**69**	4(2)
16	71	**57.1**	−1392.7	0	**71**	**35**	1(0)
Sum:	**2388**	**1370.5**		**90**	**2298**	**1054**	**115(62)**
Additional LG							
17	11	**5.8**					
18	7	**7.0**					
19	5	**9.8**					
20	4	**2.1**					

LG: Linkage Group, Mno: marker numbers on each LG, Length: length of the LG [cM], LogL: log likelihood for the given order, NoM: non-misplaced marker numbers (located on different chromosomes on the physical map), Loci: number of shared by one or several markers on the same chromosome but with different locations on the physical map, devM: observed markers on the genetic map, deviated by over one million base pairs (bp) on from the expected order in the physical map; in brackets marker numbers deviated by over two million bp (see text for details).

The number of SNPs which were supposed to map to other chromosomes on the reference map were determined. A total of 90 “misplaced” markers (3.8%) were identified in this way, varying between 0 and 14 SNPs for individual linkage groups. These markers did not affect the total lengths of the linkage groups, since they were not located at the distal ends. However, the remaining marker numbers were slightly reduced to 2298 as shown in Table 3 (column “NoM”). The remaining markers had all distinct locations on the physical map, but revealed in many cases for adjacent markers identical loci on the linkage map, since no recombination was detected between them. Table 3 shows also the number of loci which were obtained in this way for each LG. The total number of loci on the linkage map was 1,054 and they varied between 35 and 122 for the individual linkage groups (Table 3).

**Figure 1:**
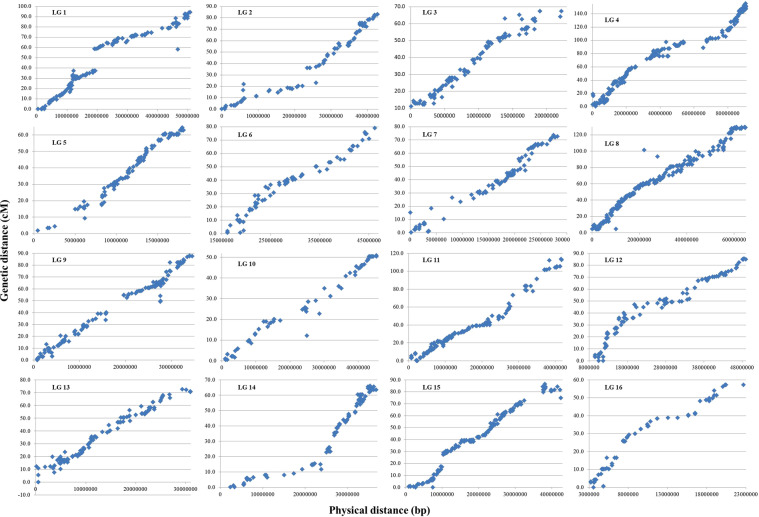
Visualization of expected and observed marker orders within linkage groups (LG). x-axis: location on physical map (bp), y-axis: location on the genetic linkage map (cM).

We also analysed the expected order of markers within linkage groups. Frequently we observed smaller rearrangements between adjacent markers, but occasionally also some larger ranking differences for certain markers. Figure 1 visualizes the differences between observed and expected marker orders for each LG considering all 2,298 SNP markers. In general, there was a good agreement since markers followed a putative regression line on each chromosome. However, also some outliers could be detected, which were located clearly apart from these regression lines. This was for example the case for one isolated individual SNP on LG1, three SNP on LG8, LG9 and LG10 and several other ones on different LG. Also larger gaps can be observed in Fig. 1: more vertical gaps such those in LG1 or LG15 represented an increase in recombination events for markers which were closely located on the physical map, while more horizontal gaps, like those which could be seen in LG4, LG5 and other LG indicate a low SNP coverage in the particular genomic regions.

**Figure 2:**
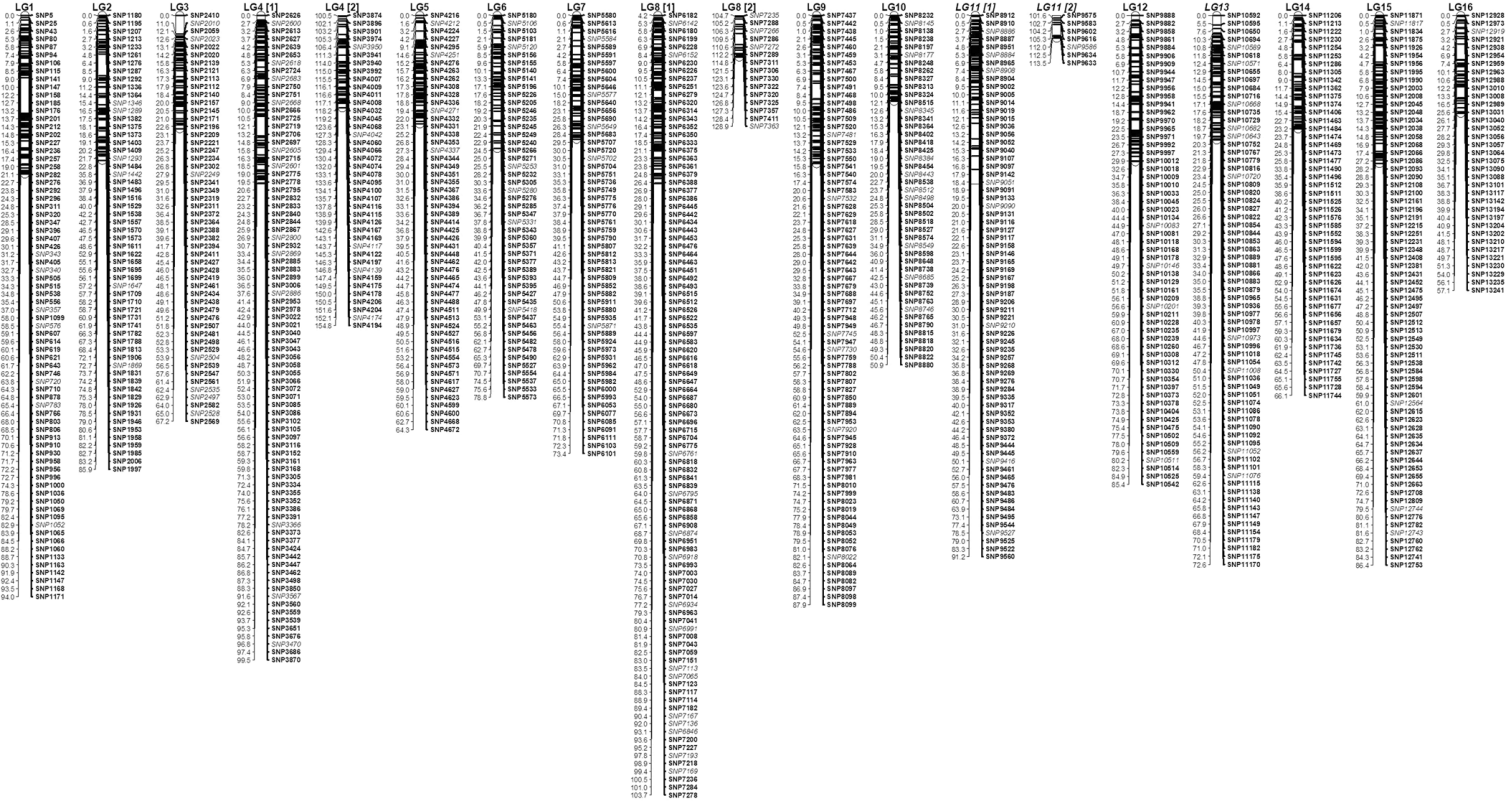
Linkage Map of the controlled cross of two oil palm genotypes obtained by Lep-MAP3. One marker is displayed per locus. SNP marker nomenclature is supposed to increase along the chromosomes. SNP markers which are shown in italics and not in bold, deviate in contrary sense to the previous marker by over one million bp.

Figure 2 presents the obtained linkage map considering only one marker per locus. Since the SNP were numbered consecutively according to their genomic location on the physical map, their SNP numbers should increase along the chromosomes. Smaller rearrangements can be identified, if consecutive SNP numbers decrease (for example SNP185/176 on LG1 at 12.7 and 13.2 cM, respectively).

Adjacent markers with over 1 million bp differences in their physical orders are indicated in italics and not in bold. The number of markers deviated in this way from their expected order are also indicated in Table 3 in the column “devM” for each LG. The first number indicates the number of markers deviated by over 1 million bp. A total number of 115 markers (11%) showed this deviation. They range from 0 markers for LG14 to 19 markers for LG8. The numbers in brackets in the column “devM” indicate the marker numbers which were deviated by over 2 million bp. The total number was reduced to almost 50% (62 markers, 5.9%) and nine LG had less than four deviated markers.

## Discussion

The SPET approach has been used up to now successfully for biomedical applications, for genotyping in maize and black poplar and for characterizing large germplasm collections of tomato and eggplant. This is the first study which applies this technology for constructing a linkage map in oil. Genetic linkage mapping in controlled crosses is based on recombination frequencies (RF) between adjacent markers. Linked markers are also expected to be in linkage disequilibrium (LD) used generally in population analyses, according to the similarity of the formulas to calculate RF and LD.

A missing data threshold of 20% was used in our study, but the values were imputed using Beagle 5.0 Software which has a very low error rate <1%^21^. Obviously the quality of a map will be better with less missing values, but also Astorkia *et al*.^22,23^ published association mapping studies in oil palm using a missing value rate of 20%.

According to Scheben *et al*.^24^, the single primer enrichment technology combines in a single approach both, targeted analysis of SNPs, thus being comparable with genotyping arrays, and complexity reduction typical of GBS approaches. Furthermore, SPET provides the ability of multiplexing thousands of samples in a single sequencing run, which can be genotyped with tens thousands of probes and with a good coverage at target sites. Finally, thanks to the sequencing of the genomic regions around the target SNPs, SPET allows the discovery of thousands of novel SNPs not originally included in the panel^20^. Also, in our study, on average 2.9 SNPs were detected per designed probe.

Other Next Generation Sequencing (NGS) technologies have been applied for linkage map construction previously. Carrasco *et al*.^25^ reported the construction of high-density linkage maps of Japanese plum (*Prunus salicina* Lindl.) using SNP markers, obtained with a GBS strategy. The consensus map was built using 732 SNPs which spanned 617 cM with an average of 0.96 cM between adjacent markers. Using SNP-GBS markers, Su *et al*.^26^ published a similar approach for maize and Gutierrez-Gonzalez^27^ for wheat. Tang *et al*.^28^ presented a high-quality genetic linkage map of 2572 SNP markers for *Stylosanthes guianensis* using the amplified-fragment single nucleotide polymorphism and methylation (AFSM) approach. Yi *et al*.^29^ used a similar technology, specific length amplified fragment sequencing (SLAF-seq) to construct an SNP-based high-density linkage map for flax (*Linum usitatissimum* L.), and Zhang *et al*.^30^ used RAD markers to construct a high-density map in channel catfish. Compared to these approaches, SPET markers have the great advantage that the specific SNP probes can be designed exclusively in coding regions as in our case. Moreover, specific genomic regions of interests can be considered in detail for targeting, for example promoters or other regulatory regions of a gene.

In the case of the oil palm, Bai *et al*.^14^ reported the largest saturated linkage map with 10,023 genome-wide SNP markers obtained by RAD-seq. A total of 5,727 SNPs were located in genes. More recently, Xia *et al*.^15^ mapped even 249,457 SLAF tags, representing an optimized version of ddRADseq, to the reference oil palm genome from MPOB using 200 individuals, but only 5064 SNPs were located within genic regions.

According to Machida-Hirano *et al*.^31^ a major limitation of restriction enzymes based methods is that they scan the genome at random loci. However, for many applications fine mapping, genotyping at specific loci, genes, or genomic regions is more critical. In this sense the valuable resources generated in the studies above can be exploited for fine mapping in specific QTL regions for traits of interest in the future.

Probe-based targeted sequencing represents a promising alternative to RADseq.^32^, and according to Mamanova *et al*.^33^ target enrichment is a feasible way to bring the field of genomics into smaller laboratories. Previous technologies for genotyping at specific target regions were not as cost effective as RAD-seq techniques, since library preparation was more laborious and time consuming, and only provided limited multiplexing options^31,32^. However, SPET is mainly PCR based, reducing considerably the costs in this way^20^.

Capture techniques are useful for the construction of dense genetic maps since sequencing will reveal all SNPs in the contig area for which the probes have been designed^16^. The SPET approach resembles a custom-made SNP array which has been used for example by Cui *et al*.^34^ to produce a high-density genetic map based on a Wheat 660K SNP array or by Joshi *et al*.^35^ for constructing a high-density linkage Map in Nile Tilapia using a 58K SNP array. Compared to these array technologies, PCR based SPET does not require the development of a new, custom-made hardware device such as a chip which is used for a reduced sample size of only 96 genotypes as in our study.

A large portion of non-segregating probes were observed in our mapping population (75%). This finding is not surprising since the SNPs were obtained from shot-gun sequencing of a panel of different *Elaeis guineensis* and *E. oleifera* genotypes. Thus, many detected SNPs could be specific for *E. oleifera* and would not segregate in *E. guineensis*. This reduced polymorphism is in part compensated by many additional SNP which were found around the target SNPs. Nevertheless, 2388 out of 3267 segregating SNP (73%) could be placed on the linkage map in this study. We have used Lep-MAP3 Software to construct our linkage map. The algorithms of Lep-MAP3 can analyse large marker numbers, but also low-coverage datasets and reduce data filtering and curation on any data. This yields more markers in the final maps with less manual work even on problematic datasets^36^. Bai *et al*.^14^ obtained a linkage map of 1,480 RAD markers used to identify interesting QTL for oil content traits. Comparing techniques and the number of markers (3,501 SPET markers), we believe that the SPET approach should be sufficient for our purposes.

In this study we have used only one panel of 5K loci for the analyses. However, it is worth to mention that these analyses could be extended to additional 5K pools, depending on the number of available SNPs. Also, other oil palm genome assemblies and SNPs such as available at Malaysian Palm Oil board^2,37^ could be considered. In this sense, our study represents more a proof of concept approach about the suitability of SPET markers for linkage map construction. It is independent of the particular reference genome used and the concrete target genes identified by the approach and could probably also be extended to other species. One straightforward application of the obtained linkage map is the exploitation for QTL analyses of new traits of interest. However, the progeny has been planted in 2018 in the field and flowering is expected for 2021, when trait recording could be initiated.

In our study we observed a total of 3.8% of markers which were misplaced on our linkage map, according to their genomic location on the physical map. Also, for some other SNPs the expected marker order deviated considerably from the expected locations on the linkage groups. It is difficult to say if this was due to scoring errors of the molecular data for linkage map construction, or due to errors in the assembly of the genome sequences. In any case, these discrepancies should be exploited for revising carefully potential alternatives in genome assemblies leading to a comprehensive validation and refinement of *de novo* genome assemblies. Unlike simply genotyping individual accessions, the expected segregations in a controlled cross are known for a given parental configuration as shown in Table 2. Thus, potential scoring errors are directly visible, and in fact we have observed this in several cases in our study. No technology is perfect and data analyses have to scope with such errors, as done by the algorithms of Lep-MAP3.

Nevertheless, even for the results obtained with only one single 5K panel, there should be sufficient markers to detect QTLs for traits of interest, in order to identify in their neighbourhood potential underlying genes with a relevant biological meaning which could explain a specific QTL. This is particularly the case when applying interval mapping techniques for QTL detection^38^, since problem of miss orders of markers can be circumvented with this more local approach. Programs such as FastQTL are able to handle large amounts of markers for this purpose^39^. Considering that often several SNP markers with different genomic locations map to the same locus in the obtained genetic linkage map, this information should be used to revise the whole genomic region for potential candidate gene detection. Besides, the density of the bait design nearby known QTL can be very easily adapted^16^. This “capture-assisted QTL mapping” of important phenotypic traits is a parallel utility^40^ and represents an advantage for breeding purposes.

Our results demonstrate that SPET represents a valid alternative to random complexity reduction methods such as GBS and micro arrays; it allows users to customize the panel of target markers and provides also a reliable technique for producing genetic linkage maps from controlled crosses. This study differs from other linkage map construction studies in the oil palm, due to the novel idea that implies the use of targeted high-throughput SNP markers for map construction which could be also affordable for modest laboratories with limited infrastructure. The map can be the first step for future analysis of quantitative trait locus of interesting traits for crop breeding. Once QTL regions have been identified, the map can be saturated with new specific probes, localized in these QTL regions for fine mapping of loci controlling specific traits.

## Materials and Methods

### Plant material

The genetic linkage map was derived from a controlled cross between a wild Dura accession from East Cameroon (Cam08) and an advanced Pisifera breeding clone from Nigeria (P320/23). A total of 94 Tenera palms from this cross as well as the two parents were genotyped using SPET Technology.

### Generation of SPET markers

Genomic DNA was obtained from around 50 mg of young leaves following the protocol of innuPREP Plant DNA extraction kit (Analytic Jena, Germany). Quality and quantity of DNA from parents and progeny samples were verified via agarose gel and Qubit fluorometer (Life Technologies). Library preparation and sequencing was performed by IGA Technology Services (IGATech Udine, Italy), using a NextSeq. 500 sequencing platform (Illumina, San Diego, CA, USA) in single-end mode (150 bp).

SNP data were retrieved from the Oil Palm Research Project (OPGP)^41^ and the whole genome sequence of the dura DELI genotype PO4906D from the OPGP project was used to design the probes for the single primer enrichment technology (NuGen. San Carlos CA, United States). A 5K panel of intragenic SNPs was selected, thoroughly covering the sixteen chromosomes of oil palm, since the main purpose was to construct a linkage map. This panel was employed to mine alleles of the genotyped parents and progeny genotypes using the Allegro Targeted Genotyping V2 procedure (IGATech Udine, Italy).

### Sequence data processing and linkage map construction

VCFtools^42^ was used for filtering the VCF file delivered by the service provider for missing value ratios and biallelic single nucleotide polymorphism (SNP) states. Beagle 5 Software^43^ was applied for imputing missing values in the filtered VCF file. For linkage map construction, Lep-MAP3 Software^36^ was applied which can handle large number of markers. The imputed VCF file was used for this purpose, together with a “pedigree file” indicating the parents of the controlled cross. The analyses were performed on a UNIX computer. The process involved several steps starting with ParentCall2 which was used to call (possible missing or erroneous) parental genotypes, followed by the Filtering2 step which filtered markers based on high segregation distortion. A p-value of 0.001 was used as threshold for the Chi-square tests. The next module was SeparateChromosomes2 which assigned markers into linkage groups (LGs) by computing all pair-wise LOD scores between markers and joined markers with LOD score higher than a user given parameter. The commonly used LOD score of 5 was used for this purpose. The JoinSingles2All module assigned singular markers to existing LGs by computing LOD scores between each single marker and markers from the existing LGs. This module generated a new map file with additional markers assigned to linkage groups. OrderMarkers2 ordered the markers within each LG by maximizing the likelihood of the data for alternative orders. A total of 100 iterations per LG were used to obtain the final map. Marker distances were expressed in cM using the Kosambi Mapping function^44^. Office software was used to extract and combine the information generated by the different output files of Lep-Map3.

Marker locations on the genetic map in cM and on the physical genome in base pairs (bp) were compared based on the original locations of the SNP markers on the 16 pseudomolecules of the mentioned OPGP genome.
